# Refining skin lesions classification performance using geometric features of superpixels

**DOI:** 10.1038/s41598-023-38706-5

**Published:** 2023-07-15

**Authors:** Simona Moldovanu, Mihaela Miron, Cristinel-Gabriel Rusu, Keka C. Biswas, Luminita Moraru

**Affiliations:** 1 Department of Computer Science and Information Technology, Faculty of Automation, Computers, Electrical Engineering and Electronics, Dunarea de Jos University of Galati, 47 Domneasca Str., 800008 Galati, Romania; 2 The Modelling and Simulation Laboratory, Dunarea de Jos University of Galati, 111 Domneasca Str., 800102 Galati, Romania; 3 Iorgu Iordan Secondary School, 125, 1 Decembrie 1918 Street, 805300 Tecuci, Romania; 4 Department of Biological Sciences, University of Alabama at Huntsville, Huntsville, AL 35899 USA; 5 Department of Chemistry, Physics and Environment, Faculty of Sciences and Environment, Dunarea de Jos University of Galati, 47 Domneasca Street, 800008 Galati, Romania

**Keywords:** Cancer, Mathematics and computing

## Abstract

This paper introduces superpixels to enhance the detection of skin lesions and to discriminate between melanoma and nevi without false negatives, in dermoscopy images. An improved Simple Linear Iterative Clustering (iSLIC) superpixels algorithm for image segmentation in digital image processing is proposed. The local graph cut method to identify the region of interest (i.e., either the nevi or melanoma lesions) has been adopted. The iSLIC algorithm is then exploited to segment sSPs. iSLIC discards all the SPs belonging to image background based on assigned labels and preserves the segmented skin lesions. A shape and geometric feature extraction task is performed for each segmented SP. The extracted features are fed into six machine learning algorithms such as: random forest, support vector machines, AdaBoost, k-nearest neighbor, decision trees (DT), Gaussian Naïve Bayes and three neural networks. These include Pattern recognition neural network, Feed forward neural network, and 1D Convolutional Neural Network for classification. The method is evaluated on the 7-Point MED-NODE and PAD-UFES-20 datasets and the results have been compared to the state-of-art findings. Extensive experiments show that the proposed method outperforms the compared existing methods in terms of accuracy.

## Introduction

Worldwide, malignant melanoma represents around 1% of all skin cancers, but if caught and detected early a timely treatment for invasive carcinoma of the skin could avert around a quarter of all deaths. Prevention and early treatment are critical. Only in 2022, about 99,780 new melanomas were diagnosed and about 7650 people died of melanoma in the United States (https://www.cancer.org/cancer/melanoma-skin-cancer). If it is detected at an early stage, a simple excision can be enough and may help the patient avoid complex therapy. Statistics show that 90–95% of patients will have a complete recovery if the melanoma is removed when its thickness is less than 1 mm. Scientists have integrated clinical methods such as the ABCDE rule or pattern recognition protocols to detect melanoma^1^. In recent years, tools belonging to the intelligent artificial techniques and imaging technologies have become more widespread, they are transforming melanoma diagnosis and improve the detection of dangerous skin lesions. Recent advancements in noninvasive diagnostic modalities provide valuable monitoring methods for persons at high risk of melanoma.

Medical image segmentation methods have broadly been used in various medical research areas and practices, dermoscopy being one of them. A SP defines a group of pixels which share similar characteristics. SP-based segmentation is widely used in many computers vision and image processing tasks as it provides a convenient use for local image features detection. The SP segmentation categorizes pixels into homogeneous regions of coherent pixels. These regions consider the object of interest, contours and may enhance the segmentation accuracy^2,3^. Moreover, SPs keep the image geometry, shape of the objects in the image with their shapes following existing contours. Many SP algorithms have been proposed. Among these, the graph-based modelling of superpixels and superpixel clustering based segmentation are the most widely known. When image boundaries are crucial for further applications, the graph-based methods are recommended. The graph-based approaches treat each pixel as a node in a graph^4,5^. The clustering-based algorithms group pixels into clusters and iteratively refine them until some convergence criteria are satisfied^4,6^.

For reliable segmentation results, pre-processing operations were strongly recommended^7^. In the case of dermoscopic images containing hairy skin lesions, the unwanted artifacts, such as hairs were removed using the Dull Razor technique. The Dull Razor algorithm replaces the hair pixels with neighboring pixel intensity values.

The pre-processed images are adopted during the segmentation stage. The simple linear iterative clustering (SLIC) algorithm is an accurate image partition algorithm which provides nearly uniform SPs. A segmented SP contains pixels that are roughly identical in characteristics and belongs to the same class. Also, these SPs do not contain edges^2,8^. One of the major drawbacks of this method is that these SPs exist in the background. These background SPs have no informational content but increase the computational load.

Algorithms for feature extraction are key tools used by dermatologists to attain accurate detection of skin cancer^9^. The extracted features from a skin lesion can describe an image either locally or globally. Feature extraction aims at extracting the features from the lesion image to characterize the melanoma. A global feature is extracted from the entire lesion or image and sometimes, it contains the background information. A local feature is extracted more densely, and it processes information from the local patch along with different parts of the lesion. Cues such as irregular distribution of color, texture and/or shape features could characterize dermoscopic images. By reviewing the existing literature, the geometric features are used more often than color and texture features^8–13^. Geometric features describe the structure and size of an object of interest by means of the perimeter, area, or its geometric shape.

Advanced image analysis tools belonging to machine learning (ML) or deep learning (DL) can objectively and consistently characterize various contents of interest in various digital image types. Algorithms for skin lesions detection, pattern recognition and classification are key components for better melanoma detection, segmentation, and classification results^14–21^. However, even state-of-the-art deep learning algorithms can fail to reach high accuracy, especially when clinical images with illumination variations, less detailed information or artefacts, and a lower contrast are used. Additionally, their performance is essentially dependent on the quality and quantity of training data and the computational cost could be a main limitation for real-time applications. Image classification is a focus point in computer vision and pattern recognition and many recent studies reported results provided by conventional techniques, transfer learning, DL, ML, and neural networks (NNs) and often combinations of them. Many SP segmentation algorithms have been proposed in the literature to deal with skin lesion classification on dermatoscopic images. Usually, the results of segmentation are evaluated by Dice and Jaccard indexes^22–27^. Different DL architectures and NN models have been recently applied to skin lesion segmentation and classification^28–33^. Sornapudi et al*.*^22^ proposed a nuclei-based segmentation method applied on histopathology images through the generation of SPs and using DL techniques. The original images are over-segmented by generating SPs and the CNN can then better identify the features in the training phase. An accuracy of 95.97% was reported. Annaby et al*.*^23^ combined the SPs graph-theoretic representations and features provided by this approach with some traditional dermoscopic image features based on color, geometry and texture to enhance the detection performance. Several classifiers were trained and tested on different combinations from those features and an accuracy of 97.40%, a specificity of 95.16% and a sensitivity of 100% were reported in the case of melanoma detection performance. Huang et al*.*^24^ proposed a spatial relationship technique among SPs with KNN to reclassify and avoid misclassification in breast lesions diagnosis. A CNN and a distance metric learning-based classifier are used to classify the SPs. Various regions of interest such as fat, mammary, tumor, muscle and chest were analyzed. The authors reported improved performance against the well-known methods as UNet, SegNet, PSPNET etc., even with a small amount of breast images. Moldovanu et al*.*^25^ proposed a melanoma classification method based on 2D Higuchi’s fractal surface features. Images belonging to three databases were analyzed. The results showed that the accuracy of the Radial Basis Function Neural Network is higher than 94% for all databases. Afza *et al.*^26^ proposed a hierarchical framework based on SPs and deep learning. They enhanced the image contrast as a preprocessing step, then the skin lesions are segmented using a two-dimensional SPs segmentation. Furthermore, ResNet-50 and Naïve Bayes classifiers were employed. The proposed method was evaluated on three databases (PH^2^, ISBI2016, and HAM1000), and they reported an overall accuracy of 95.40%, 91.1%, and 85.50%, respectively. Rout et al*.*^27^, combined the watershed transform with a clustering algorithm to extract melanocytic skin lesions from dermoscopic images. The lesion boundary regions are enhanced using a Gaussian filter and the acquired SPs are clustered using a fast fuzzy c-means clustering technique to obtain the final segmented lesion. The proposed method is tested on several databases: ISIC 2016, ISIC 2017 and ISIC 2018. An accuracy of 96.41% is obtained for a 3 $\times$ 3 Gaussian filter kernels. Deep learning architectures were used by He et al*.*^28^ to learn some contrast features to be used for salient object detection. By using the SPs mechanism, the computation complexity was reduced. Jianwu et al.^29^ studied the effect of the number of SPs on the image classification. The classification method used a graph neural network model and deep features which are extracted from images converted into region adjacency graphs which consist of SPs. Chhablani et al*.*^30^ proposed a Deep Neural Networks model based on the higher-order information provided by SPs and performed classification tasks using images from different datasets, such as handwriting recognition, from fashion and clothing domains, object recognition domains or medical images.

In addition, many methods based on raw pixels have been proposed to classify the dermoscopic images using the shape features^31–36^. Moussa et al*.*^31^ investigated the utility of certain geometric features such as the asymmetry, border and diameter in melanoma classification by using a KNN algorithm. Shetty et al*.*^32^ investigated the classification accuracy of both the ML algorithms and CNN models for pigmented skin lesions. They employed various ML models such as DT, RF, SVM, KNN, Logistic Regression (LR), GNB and Linear Discriminant Analysis (LDA). The CNN models were built using Tensorflow and Keras libraries. An accuracy of 95.18% was reported for the customized CNN, which is better compared to the used ML algorithms. In a recent study, Akram et al.^34^ used a variant of the SVM classifier to detect and classify skin lesions. Before classification, they had performed a features selection using the texture feature analysis, labels, boundary connections and central distances. Certain classifiers such as KNN, DT, RF, multi-layer perceptron and SVM were used by Janney and Roslin^35^ to classify melanoma. The features analyzed included circularity and irregularity. Hybrid features covering geometrical shape, color and texture features were introduced by Mukherjee et al*.*^36^ to improve the classification accuracy in malignant melanoma detection. The extracted features are classified using the classical SVM, KNN and Ensemble Boosted Tree classifiers. In the paper^11^, a large number of shape features, such as asymmetry, diameter, border and color were used to generate the total dermoscopic value (TDS). The classification is done by using the TDS score.

In summary, both ML algorithms and DL techniques combine both local and global features for an improved and accurate classification task. They ask for more training data or for pre-trained models but face a main limitation related to the computation complexity. The SPs approach proposed in our paper is neither local, nor does it use the global representation, but has an important contribution to the dimensionality reduction for less computation time and improved accuracy. The hypothesis that a robust and consistent SP segmentation, a performant SP selection and quantitative SP shape feature extraction can enhance the accuracy of melanoma detection and classification is employed. Also, the SPs are generated using an unsupervised method, they keep low-level details and downscale the image – with the direct consequence being the low computational cost.

This paper utilizes the SPs segmentation method for melanoma detection and classification proposed in the state of the art along with some original processing that brings some improvements in classification. A well-established local graph cut algorithm is adopted to identify the region of interests (ROIs) in dermoscopy images. An improved Simple Linear Iterative Clustering (iSLIC) algorithm is exploited to segment SPs. Specifically, iSLIC selects the SPs of interest in the segmentation map through the instrumentality of labelling and discards those SPs belonging to the background. Then, the obtained map is used for the SPs’ feature extraction to utilize the spatial context of skin lesions as additional information to improve the melanoma classification. Using SPs instead of individual pixels improves the computational efficiency and the performance of classification, as the SPs contain pixels that are largely identical in features and belong to the same class. Moreover, this approach is hybrid by nature and improves the image description through the features instrumentality. We extracted six types of features, for each SP. All the features were normalized. Then, six ML tools such as, RF, AD, DT, GNB, KNN, and SVM^14–21^. and three NNs such as, PRNN, FNN and 1D CNN were trained using the selected features to classify SPs for melanoma detection. The performance of the proposed approach is measured quantitatively based on the following metrics: accuracy, recall, F1-score, precision and Matthew’s correlation coefficient (MCC).

Our contributions compared to other state-of-the-art approaches are summarized as:

1. An improved Simple Linear Iterative Clustering (iSLIC) algorithm is proposed for melanoma and nevi classification in demoscopy images following two new visions:
  - A new algorithm which assigns labels to SPs, this allowing the background SPs to be eliminated;
  - A shape SPs analysis is proposed to generate the input vectors for classification purposes. Thus, the geometric features (perimeter, area, eccentricity, orientation, convex area, and major axis length) of the SPs were computed and fed to nine classifiers.
2. The validation framework covers tree datasets.
3. To the best of our knowledge, we are the first to apply the segmented SPs for shape and geometric feature extraction and classification task.

## Proposed framework

In this section, we describe in detail our approach for skin lesion classification.

**Figure 1:**
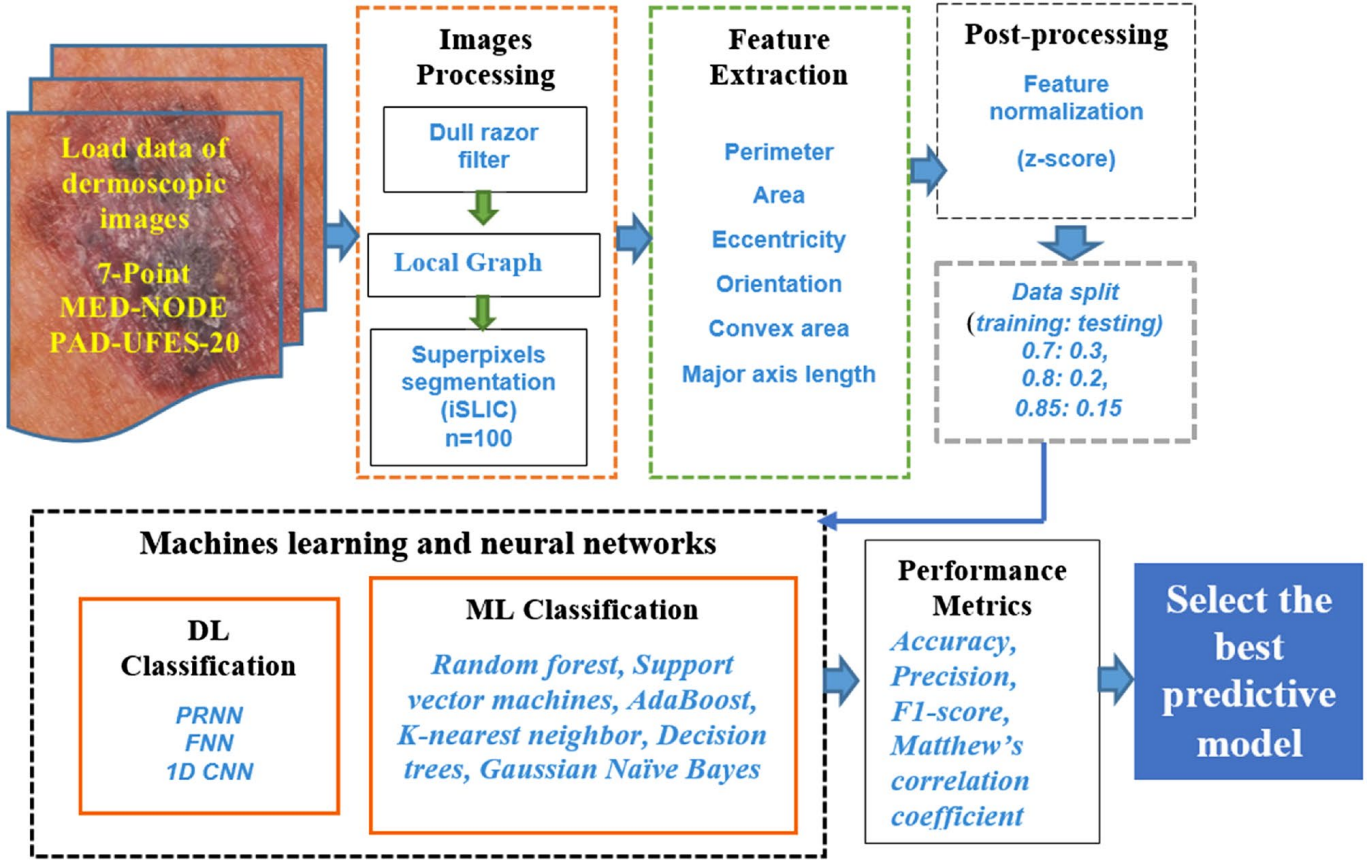
Block diagram of the proposed model for skin lesion classification. The input to the classifiers is the normalized features extracted from SPs.

A brief conceptual block diagram of our pipelines and the main stages of our research are illustrated in Fig. 1. In the first stage, the images dataset is built using images provided by the 7-Point, MED-NOD and PAD-UFES-20 databases. During the preprocessing stage, artefacts such as hair and noise are removed as they could adversely affect the segmentation performance. The segmentation of pre-processed images is performed using the graph cut local method. Because of this step, the possible hidden confounder and so-called ‘shortcut learning’ are avoided and we can focus only on analyzing the regions of interest from now on. For SPs generation, we employed the iSLIC algorithm on the identified regions of interests. This will minimize the complexity of the further processing steps. The segmented SPs generated from nevi and melanoma lesion images allow the computation of relevant shape and geometric features. The normalized features data is then fed into the MLs and NNs tools for classification.

## Results and discussion

In this section, six ML classifiers and three NN models were used to test the performance of skin lesion classification based on the features of SPs. The iSLIC segmentation method explores the spatial correlation of pixels, exposes the low-dimensional structure of pixels and finally, improves the classification results. By using different SP segmentation numbers there is found out that n = 100 provides the high accuracy of classification at a relatively low dimension. Three datasets, named 7-Point, MED-NODE and PAD-UFES-20, are used for experimental investigation.

**Figure 2:**
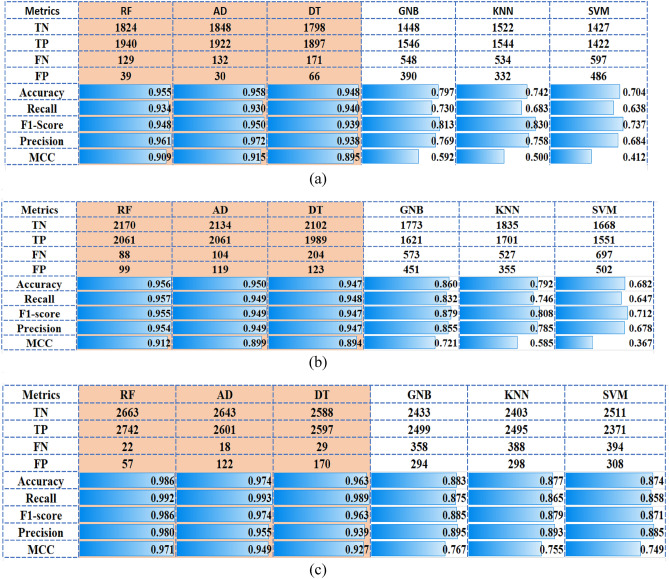
Performance plots in the test dataset, for ML classifiers. The ratio ‘training:testing’ is 0.7:0.3. The best accuracy, precision, sensitivity, F1-score and MCC scores are provided by RF, AD and DT algorithms. (**a**) MED-NODE dataset; (**b**) 7-Point dataset; (**c**) PAD-UFES-20.

**Figure 3:**
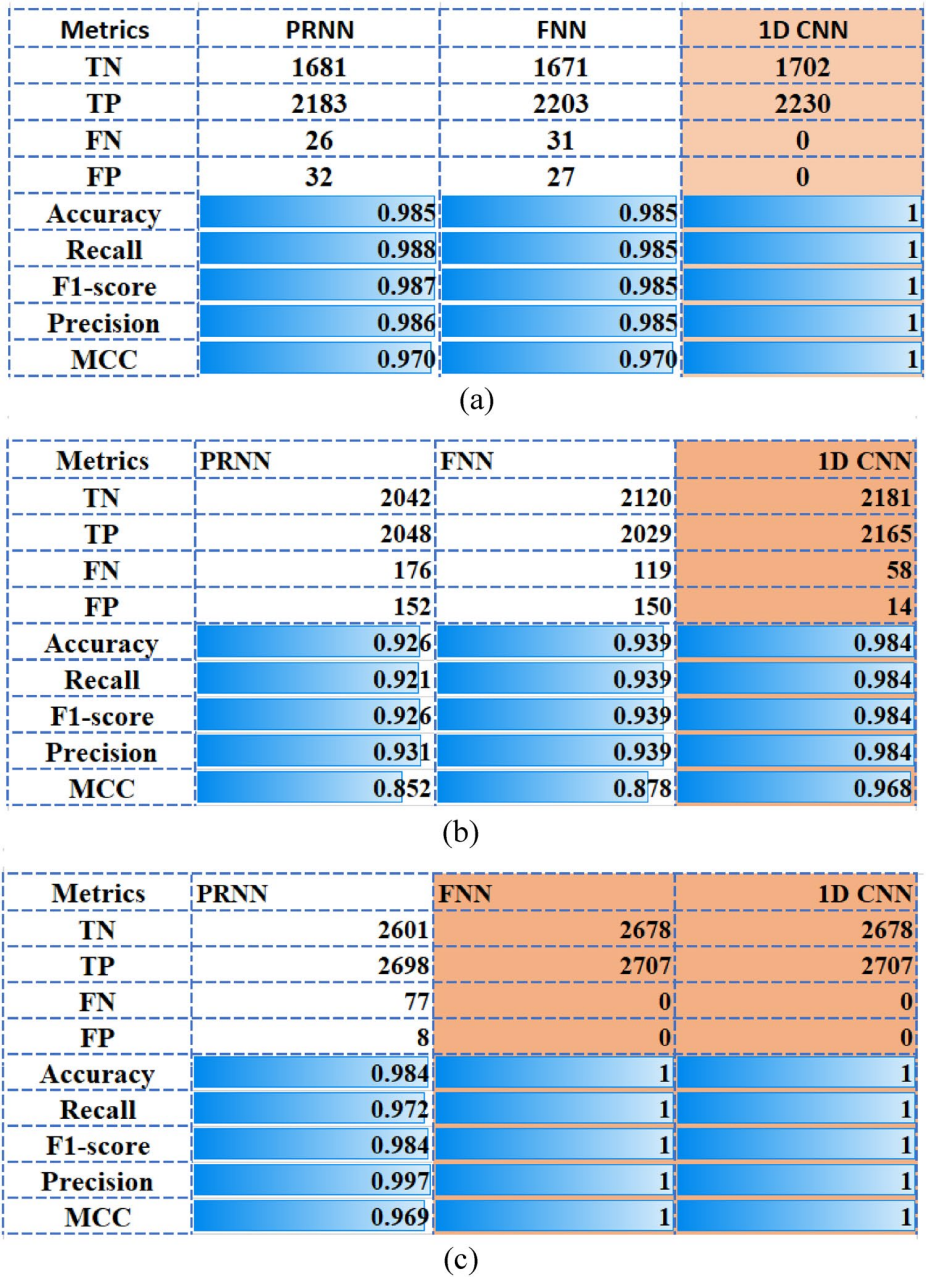
Performance plots in the test dataset, for NN classifiers. The ratio ‘training:testing’ is 0.7:0.3. The best accuracy, precision, sensitivity, F1-score and MCC scores are provided by 1D CNN model. (**a**) MED-NODE dataset; (**b**) 7-Point dataset; (**c**) PAD-UFES-20.

The SP approach is focused on the data distribution to avoid insufficient and low-quality data. Overall, the experiment indicates that the SPs segmentation approach is useful for better classification accuracy. The reason is mainly because from a small number of dermoscopic images, a large number of SPs can be generated are further used as training samples. These SPs carry both low-level details and high-level information as the pixels inside the SP do not need to be labeled. First, we have analyzed the impact of different sizes of training and testing sets. So, the SP datasets were split into training and test sets using the ratios 0.7:0.3, 0.8:0.2, 0.85:0.15 (training:testing). The best classification results were obtained for the ratio of 0.7:0.3. Also, the fivefold cross-validation has been used to prevent overfitting and to increase the accuracy of classification in the training set. The accuracy is provided as the mean of the accuracies of the fivefold models. For each classifier and dataset, the details of the confusion matrix and performance metrics are shown in Figs. 2 and 3. Figure 2 displays the best accuracy, precision, sensitivity, F1-score and MCC scores obtained for the RF, AD and DT algorithms. From Fig. 3, it is noted that the 1D CNN model outperforms in terms of accuracy, precision, sensitivity, F1-score and MCC scores the PRNN and FNN models.

Figure 2 presented the proposed classification results on the utilized test datasets (i.e., 30% of the samples in SPs dataset). The best classification accuracy was achieved by RF, AD, and DT classifiers, as the accuracy is higher than 0.95, recall, F1-score and precision achieved values higher that 0.94 and MCC values are better than 0.89, respectively. A poor performance of classification has been provided by GNB, KNN and SVM algorithms with the accuracy < 0.86, recall < 0.83, f1-score of 0.71, precision of 0.85 and MCC lower than 0.72. In Fig. 3, the performance of classification on the test datasets is provided for each NN model. It is noted accuracy (0.984), recall (0.984), F1-score (0.984), precision (0.984) and MCC (0.968) are achieved by the 1D CNN classifier for 7-Point dataset while the best performance is obtained for MED-NODE and PAD-UFES-20 datasets. It is important to highlight that the higher performance metric values were measured on the test set and they are not the result of overfitting. The declared goal of our research to discriminate between melanoma and nevi without false negatives was achieved through the use of 1D CNN. This model classifies all the positive samples as positive, and does not misclassify a negative sample as positive in the case of MED-NODE and PAD-UFES-20 datasets.

The achieved accuracy of the proposed method is sufficient for a fair comparison with other published techniques. In the cases of RF, DT and AD algorithms improved results are reported, while the SVM classifier provided good results that are in line with other reported results. In our experiment, the SVM classifier is less performant. Table 1 presented the achieved performance of a few relevant methods. Some authors used only one ML tool while other authors used combined tools. The researchers used classifiers such as SVM, KNN, DT, and RF for melanoma diagnosis with accuracies ranging from 48.39% to 92.1%, respectively. Generally, the SVM classifier gives the best accuracy.

**Table 1:** Summary of classification techniques from previous studies.

	Metrics	
	Sensitivity	Accuracy
Giotis et al*.*^37^	–	92.1% (SVM)
Moussa et al*.*^31^	–	89% (KNN)
Shetty et al*.*^32^	90% (AD)	53.12% (SVM) 48.39% (KNN) 68.66% (DT) 87.32% (RF) 36.33% (NB)
Janney and Roslin^35^	93.97% (RF)	58% (KNN) 78% (DT) 86% (RF)
Mukherjee et al*.*^36^	–	86.1% (SVM)
Chhablani et al.^30^	–	99.30% (CNN), 99.21% (CNN + GNN)
Shetty et al*.*^32^	–	86.43% (CNN, test set)
Khan et al*.*^46^	81.24% (NB) 85.42% (MSVM) 82% (fine KNN)	81.34% (NB) 85.50% (MSVM) 82.08% (fine KNN)

The classification of skin lesion images is a very challenging task, mostly due to the low contrast which makes it difficult to differentiate the border of the lesion or due to the need of extensive training, small interclass variation, multi-shape images, and imbalanced classes of datasets. The last issue is a critical one and usually, the performance of the SVM and KNN classifiers is strongly influenced. These algorithms ask for very large data sets^47^. In spite of the fact that SPs dramatically increase the number of input samples in our experiment, the performance of classification for SVM, KNN and GNB is lower than before but generally still satisfactory.

Also, various DL models are employed by researchers to acquire good performances. There is a noticeable trend in the number of studies using DL for melanoma detection. When a large number of images is provided, DL yields superior performance. In this case, the utilization of DL techniques asks for precaution as the datasets often have insufficient samples to allow sound learning of characteristics that vary significantly. The issues of less training data, overfitting and/or underfitting of the model cause misclassification and reduce the effectiveness of the approaches. However, NN models are more appropriate to solve the classification problem and they can even outperform other traditional techniques. When the problem of insufficient training data is overcome, the accuracy of classification is a little bit higher than machine learning approach. It is worth mentioning that, in the proposed work, the number of generated SPs allow for balanced classes of datasets and lead to a strong generalization. The results show that the 1D CNN has obtained an accuracy of 100% for MED-NODE and PAD-UFES-20 datasets and 98,48% for 7-Point dataset, which is better than the utilized machine learning algorithms.

The proposed method has some limitations. The SLIC superpixels segmentation algorithm is a subject to boundaries preservation. It is well-known that the boundary is subject to the performance of the SP segmentation algorithm. This issue has been overcome by proposing the improved SPs segmentation algorithm, namely iSLIC. The results are very good as the dermoscopic images suffer itself from low contrast which makes it difficult to differentiate the border of the lesions. Another limitation consists of the heuristic approach through which the CNN architectures were developed. The CNN was trained and progressively refined by changing the hyperparameters until this was proven experimentally to improve the detection rates. A pre-trained CNN model didn’t find applicability in this study because it is capable of discriminating images of different objects classes, but may be less effective in discerning the difference between different textures in the same object. In future works, the knowledge acquired in this stage should be used from the beginning in the handcrafted deep convolutional networks architecture design.

## Conclusion

This study proposes to classify skin lesion image by using the SP features. The goal was to find the best intelligent artificial tools in terms of performances capable to differentiate skin lesions. Instead of extracted features from images we proposed a SPs approach for feature extraction. The SPs were generated using an improved Simple Linear Iterative Clustering (iSLIC) and shape and geometric features were gathered from each segmented SP. Six Machine Learning algorithms and three Neural Networks models were used for classification purposes and the best model for skin lesion classification was determined to be the 1D CNN. This model is a better alternative to ML techniques by providing better results overall. The SPs approach strongly increases the number of input data for classification so that more SPs, the higher the classification accuracy is obtained. Finally, comparative experiments demonstrate that our proposed method outperforms the compared methods in terms of accuracy. The proposed method explores a more practical approach as it is able to pick useful features from multiple regions containing groups of pixels that look similar but belonging to the same skin lesion image. In this way, the classification overcomes the shortcomings of under-represent darker skin types, as an example. This actually ensures the independence of the proposed method from the diversity of images in datasets.

In future works, we intend to test the proposed method on hybrid features based on shape, color and texture and also to use more image databases.

## Methods

### Databases

Three image databases, 7-Point MED-NODE and PAD-UFES-20, are evaluated in the experiments. Class distribution statistics of datasets are provided in Table 2.

**Table 2:** Class distribution statistics of datasets.

Datasets	No. of images	No. of classes	No. of attributes	No. of nevus/melanoma/ atypical nevus	Image type
MED-NODE	170	2	4	100/70/0	Non- dermoscopic^37^
7-Point	439	3	4	68/297/74	Dermoscopic^38^
PAD-UFES-20	289	2	4	241/48/0	Dermoscopic^39^

The Algorithm 1 (n = 100) runs for each dataset and augments the number of processed objects, as follows:

- MED-NODE: 170 images generate 13,073 SPs;
- 7-Point: 200 images generate 14,726 SPs.
- PAD-UFES-20: 289 images generate 17,948 SPs.

### Algorithm 1: Superpixel segmentation and geometric features extraction

Algorithm 1 performs a robust and consistent SP segmentation, a performant SP selection and quantitative SP shape feature extraction. The proposed iSLIC selects the SPs of interest in the segmentation map and further, this map is used for the SPs’ feature extraction. iSLIC allows to assess multiple regions containing groups of pixels that look similar, from the same image, for diagnosing melanoma. In order to test algorithm generalizability, various classifiers are used. Algorithm 1 generates SPs from the image datasets and allows the shape and geometrical features extraction. The normalized features feed the MLs tools (RF, AD, DT, GNB, KNN and SVM), and neural networks (PRNN, FNN and 1D CNN) in order to output the category for each SP. The *superpixels_procedure()* contains the iSLIC algorithm and *properties_procedure()* includes the tools for perimeter, area, eccentricity, orientation, convex area and major axis length computation. These features are denoted as Ai, Pi, Ei, Oi, ENi, CVi. The variable n is the number of SPs intended to be generated (n = 100), Li is a label matrix of type double and Ni is the number of SPs that is computed. To discard the SPs that belong to the background, a black mask is generated. In the structure *if…then,* the conditionality as only that labelled SPs are kept is imposed. These SPs belong to the mask. The rest of the SPs are discarded.
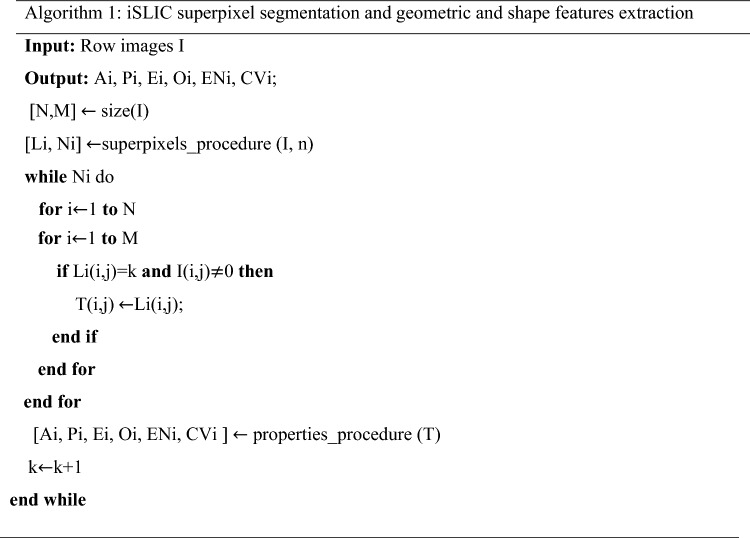


The proposed method is implemented in Python 3.8.16 via Google Colab IDE and Matlab R2018a. The ML classifiers used the Scikit-learn library for machine learning and the neural networks used the Keras library from the TensorFlow framework. The features of the SPs are analyzed using the Image Processing toolbox. The System has the configuration of Intel(R) Core (TM) i7-8550U CPU @ 1.80 GHz 1.99 GHz—a Windows 10 (64 bit) machine with 8 GB of RAM.

### Splitting dataset and data normalization

The SPs data generated from the 7-Point, MED-NODE and PAD-UFES-20 databases is split into training and test sets using the ratios, 0.7:0.3, 0.8:0.2, 0.85:0.15 (training:testing). These ratios allow the models to learn and adapt to various scenarios and to overcome the overfitting phenomena. To avoid data leakage, the normalization process is applied on the partitioned data into both the training and test sets, by using the z-score normalization^40^,

1 $$a{\prime} = \frac{a - \mu \left( A \right)}{{\sigma \left( A \right)}}$$

where $\mu$ and $\sigma$ are the mean and standard deviation values, respectively, of the vector A.* a* is each record from vector A to be normalized. *a'* is the result of normalization.

### Geometric and shape features

**Figure 4:**
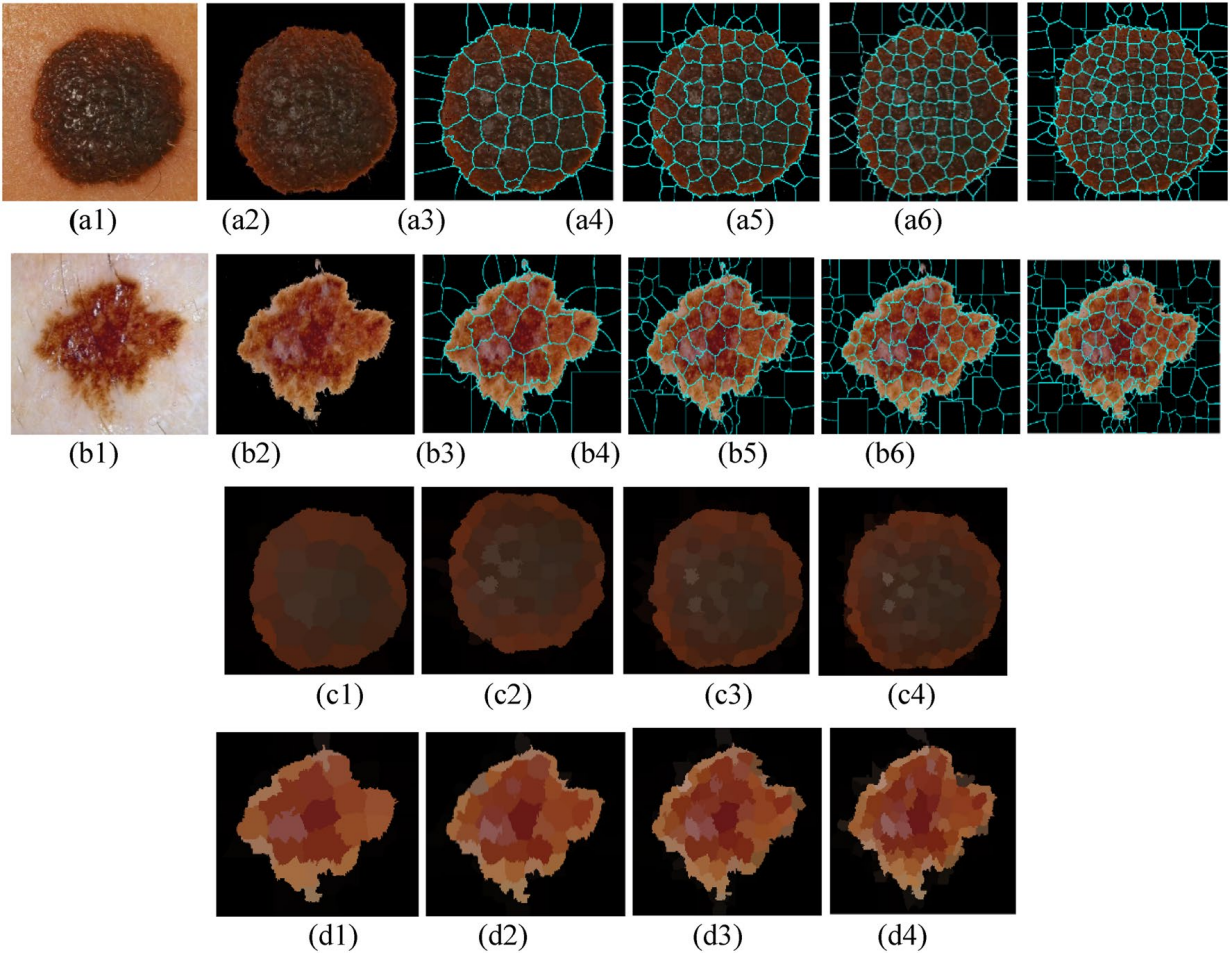
SP generation and segmentation. (**a1**, **b1**) Original images belong 7-Point dataset; (**a2**, **b2**) Pre-processed with the dull razor filter and segmented images with the black mask inserted in the background; (**a3**–**a6**) SP boundaries of nevi; (**b3**–**b6**) SP boundaries of melanoma; (**c1**–**c4**) Final segmentation of nevi where all background SPs are removed; (**d1**–**d4**) Final segmentation of melanoma where all background SPs are removed.

In this paper, the goal is to segment, based on SP computation, the skin lesion from pre-processed dermoscopic images. The segmented SPs are useful for relevant feature extraction and they keep the low-level details and downscale the image—a direct consequence being the low computation cost. Also, the SPs contain low-level information and are the perfect way to reduce the loss of details. Figure 4 illustrates the SPs segmentation results for two sampled images belonging to the 7–Point database. The first row denoted (a) shows the SPs segmentation results for nevi; the second row (b) for melanoma; rows (c) and (d) display the results of segmentation where all background SPs are removed. iSLIC generates an over-segmented image with more or less n pieces. Third to last column (i.e., a3 to a6 and b3 to b6) present the SPs segmented images for several n values (n = 50, 100, 150, 200). The impact of different SP segmentation numbers to the computational cost of classification has been evaluated. The performance of classification is not greatly affected by the number of generated SPs but the best classification results are obtained for n = 100.

The features computed in our framework include the perimeter, area, eccentricity, orientation, convex area and major axis length^37^. The area and perimeter are the most relevant shape features^10–12^. Table 3 exemplifies the features extracted from a SP generated into the skin lesion.

**Table 3:** Shape and geometric features.

Features	Graphic illustation	Features description
Area		The area is the number of pixels in a region. It is marked in black
Perimeter		The perimeter is distance between each adjoining pair of pixels around the border of the SP. It is marked in black
Orientation		The orientation is the angle between the *x*-axis and the major axis of the ellipse fitting the SP. Its values range from − 90° to 90°
Major axis length		The length of the major axis of the fit-ellipse
Eccentricity		It is the ratio of the distance between the foci of the fit-ellipse and its major axis length. The value is between 0 and 1
Convex area		It is the number of pixels in a convex image

### Machine learning classifiers

The classification is the most important step in skin cancer recognition as it distinguishes among the skin lesions and ML algorithms perform very well in this area. However, it is difficult to offer a fair and accurate comparison among different ML methods as they used various hyperparameters, different variants and several datasets. The most popular ML techniques are AB, SVM and DT^14–20^, but KNN, RF, GNB classifiers are also mentioned regularly. This study focuses, in the first part, on the classification provided by the well-known ML classifiers—RF, AD, DT, GNB, KNN, and SVM.

- *Random forest* (RF) is a technique based on decision tree algorithms and generalizes the classification process using two types of randomizations: at the tree level and at the node level. The first ensures that each tree is fed by a bootstrap, while the second refer to a subset of feature dimensions which is randomly selected from the original dimension^14^.
- *Support vector machine* (SVM) is a linear classifier that can separate two classes by finding the maximum margin separating hyperplane between the two classes^10,15^. To return the best accuracy of classification, choosing the right kernel is essential^16,17^.
- *AdaBoost* (AD) is a meta-estimator in ML being used as an ensemble method. Usually, decision trees with one level are used with AD. AD is employed for tackling binary classification problems so it needs assign to each registration some weights. Initially, the weights are equal. For each feature, a decision stump is generated and the Gini Index of each tree is calculated. The tree having the lowest Gini Index creates the first stump^15^. The iterative process stops when a low training error is achieved.
- *K-nearest neighbor* (KNN) is a non-parametric supervised learning classifier. It classifies data based on their proximate neighbors. For a correct classification, the input parameters are K, the number of nearest neighbors and d, the distance between neighbors. The Euclidean distance, Hamming distance, Manhattan distance, and Minkowski distance are the usual distances used^18^.
- *Decision tree* (DT) is a non-parametric supervised learning method that uses hierarchical trees for classification^18,19^. DT repeatedly splits the data set according to a criterion that maximizes the separation of the data^20^.
- *Gaussian Naïve Bayes* (GNB) is a supervised learning classifier based on Bayes theorem for probabilistic classification. NB uses the maximum likelihood method to estimate the particular values for mean and standard deviation. The GNB starts with the “naïve” assumption of conditional independence between every sample of features obtained of the melanoma and nevi^20^. This assumption is not always true but using the generative learning mechanism, the models is able to predict the posterior probability.

The hyperparameters in ML classifiers that control the learning process are shown in Table 4.

**Table 4:** Hyperparameters in proposed machine learning models.

Model	Hyperparameters
RF	n_estimators = 100, *, criterion = 'entropy'
SVM	kernel = 'liniar', degree = 2, gamma = 'scale', cache_size = 100, decision_function_shape = 'ovo'
AD	n_estimators = 100 algorithm = 'SAMME', random_state = 40
KNN	n_neighbors = 1, *, weights = 'uniform', algorithm = ‘kd_tree’, leaf_size = 20, p = 2, metric = 'euclidean'
DT	criterion = 'entropy', splitter = 'best', max_depth = 100, ccp_alpha = 0.0
GNB	priors = None, var_smoothing = 1e-^09^

Where the hyperparameters are not explicitly defined, they are considerate as default.

### Neural network classifiers

Various deep learning techniques are employed to acquire relevant features in melanoma classification^26,28–30,32,41,42^. The classification was performed using the well-established and standardized databases where the images are stored in different modalities. During the classification process employing deep learning methods, the main drawbacks to be overcome are related to having access to little training data, overfitting, and underfitting of the models. Also, some challenges occur when complex and/or rare features are considered. A wide range of skin features is available to researchers. The feature extraction and selection reduce the size of the input vector for AI models and also improve the computation load. The present study uses, in the second part, three neural networks to cope with the SP features extraction.

- *Pattern Recognition Neural Networks* (PRNNs) are feedforward networks that makes decisions from complex patterns of information. In the hidden layer, the pattern that is not linearly separable is transformed into higher-dimensional space that is more linearly separable.
- *Feedforward Neural Networks* (FNNs) are neural networks with a simple architecture where the input is processed in only one direction. The node connections do not form a cycle.
- *1-Dimensional Convolution Neural Networks (*1D-CNNs) are used in the case of certain applications where they are advantageous and thus preferable to their 2D models as they have the computational complexity significantly lower, their compact configurations are easier to train and implement and are relatively quick to train^43,44^. It has an increased capability to find a function of fixed complexity that approximates the nonlinear relationships between variables.

The hyperparameter values used in the NN models are highlighted in Table 5. The selection of NN hyperparameters was performed around the values suggested by the KerasTuner, a general-purpose hyperparameter tuning library. The Adam optimization algorithm for training NNs is simple to use and computationally efficient. Binary cross-entropy calculates the loss value. The epoch count is 100 for low loss and no overfitting. The rate of learning is initially set to 0.01.

**Table 5:** The neural networks hyperparameters.

NNs	Hyperparameters
PRNN	hidden layer sizes = 10, training function = 'trainscg', Performance function = 'crossentropy', number epochs = 100
FNN	Input layer (10 neurons, activation = 'tanh'), 2 Hidden Layer (2nd layer – hidden layer with 8 neurons, activation = 'tanh' and 3rd layer – hidden layer with 6 neurons, activation = 'tanh'), Output layer (1 neuron, activation = 'sigmoid') optimizer = 'adam', learning rate = 0.001, loss = 'binary_crossentropy', metrics = ['accuracy'], number epochs = 100
1D-CNN	2 Conv1D layers, 1 MaxPool1D layer and 2 Dense Layers as follows: Conv1D layer (filters = 64, kernel_size = 2, activation = 'relu'), Dropout layer (dropout rate = 0.2), Conv1D layer (filters = 32, kernel_size = 1, activation = 'relu'), MaxPool1D layer (pool_size = 1), Flatten layer, Dense layer (32 neurons, activation = 'relu'), Dense layer (1 neuron, activation = 'sigmoid'). loss = 'binary_crossentropy’, optimizer = 'adam', learning rate = 0.001, metrics = ['accuracy']

**Figure 5:**
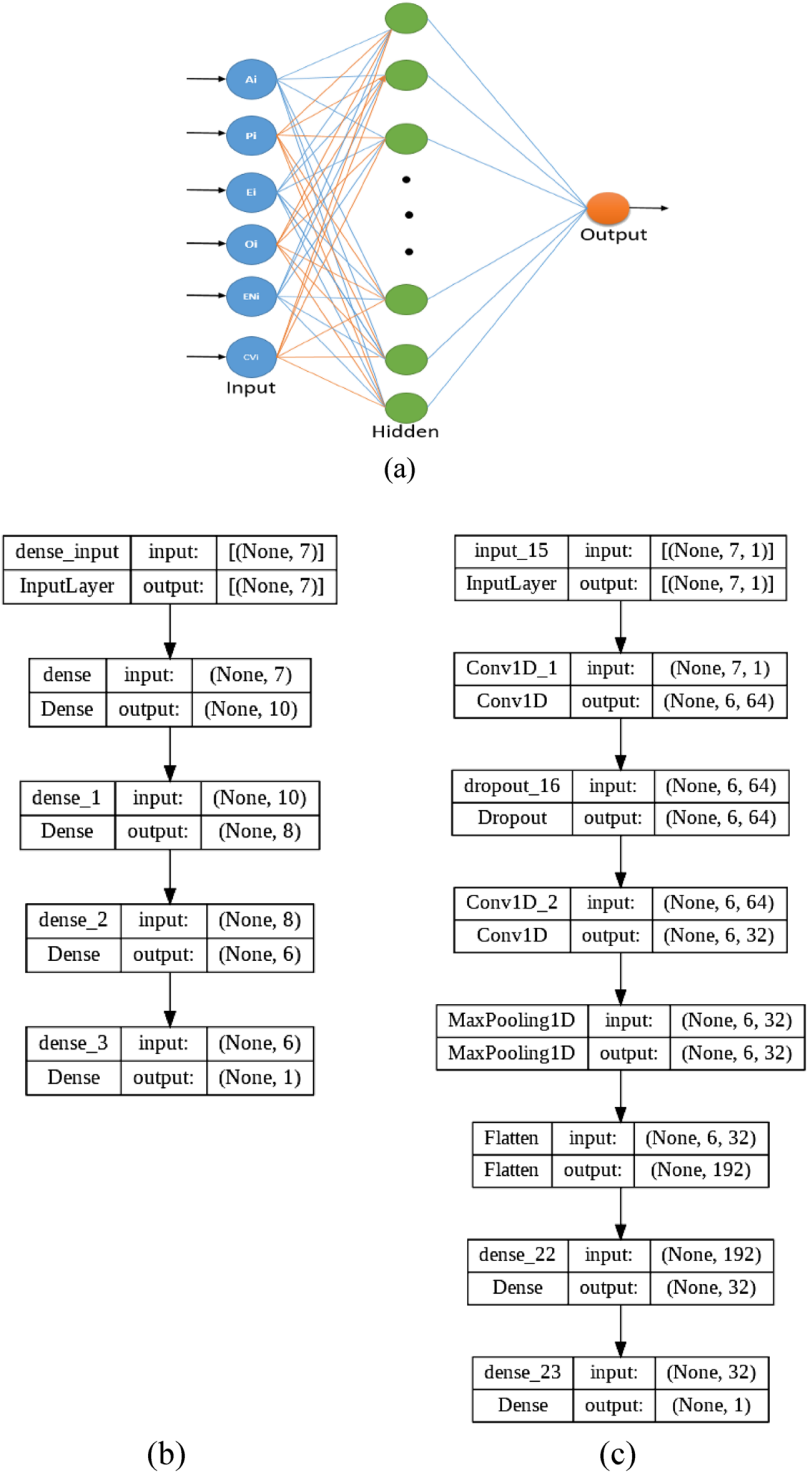
The configuration of the NN architectures. (**a**) PRNN architecture; (**b**) FNN architecture; (**c**) 1D CNN architecture with 2 CNN layers.

The configurations of the used NN architectures are shown in Fig. 5.

The following measures have been used to evaluate the performance of classification: accuracy, precision, recall, F1-score and most importantly, Matthew’s correlation coefficient (MCC)^14–20,45^. They are extracted from the confusion matrix.

Accuracy represents the number of total samples correctly predicted reported to the total number of predictions^22–25^,

2 $$Accuracy = \frac{TP + TN}{{TP + TN + FN + FP}}$$

Precision is the ratio of correctly predicted positives reported to the total number of positive cases.

3 $${\text{Precision}} = \frac{TP}{{TP + FP}}$$

The recall calculates the ratio of predicted positive samples to the true positive plus the false negative cases. It neglects how the negative samples are classified.

4 $${\text{Recall}} = \frac{TP}{{TP + FN}}$$

The F1-Score calculates the weighted average of both the Precision and Recall and can maximize either of them. A maximum F1 value indicates that the classification model has an optimal balance of recall and precision.

5 $$F1 - Score = \frac{{2 * {\text{Recall}} * {\text{Precision}}}}{{{\text{Recall}} + {\text{Precision}}}}$$

For imbalanced datasets, the accuracy and F1-score measures can provide overoptimistic, inflated results. The Matthews correlation coefficient (MCC) is used to overcome this issue. It produces a high score if the classifier correctly predicts most of the positive and negative data samples^45^. Particularly, MCC provides a correct prediction when there are many TP samples but few TNs (or vice-versa). In this case, F1-score and accuracy can provide spurious information.

6 $$MCC = \frac{TP \times TN - FP \times FN}{{\sqrt {\left( {TP + FP} \right)\left( {TP + FN} \right)\left( {TN + FP} \right)\left( {TN + FN} \right)} }}$$

Here, TP (True Positive) refers to the number of correct samples, classified as positive samples, TN (True negative) is the number of correct samples, classified as negative, FP (False positive) is the number of negative samples that are predicted to be positive and FN (False negative) represents the number of positive samples that are predicted to be negative.

## Data Availability

The MED-NODE dataset is freely for download and publicly available at, https://www.cs.rug.nl/~imaging/databases/melanoma_naevi/. The 7-Point dataset is freely for download and publicly available at, http://derm.cs.sfu.ca/. The PAD-UFES-20 dataset is freely for download and publicly available at, https://data.mendeley.com/datasets/zr7vgbcyr2/1. The datasets generated during the current study are available in the repositories, https://gitfront.io/r/mmigyt/tbKZyo8JNxgv/Binary-Classification/ , https://gitfront.io/r/mmigyt/MPRmUdFDz1qV/Binary-Classification-Neural-Networks/
